# National Trends in Cerebrovascular Disease–Related Mortality among Adults With Obesity in the United States, 1999–2020

**DOI:** 10.1002/brb3.71276

**Published:** 2026-02-28

**Authors:** Ibrahim Nagmeldin Hassan, Siddig Yaqub, Muhsin Ibrahim, Nagmeldin Abuassa, Mohamed Ibrahim, Shahzaib Ahmed, Allahdad Khan, Hamza Ashraf

**Affiliations:** ^1^ University of Khartoum Faculty of Medicine Khartoum Sudan; ^2^ Faculty of Medicine Karary University Khartoum Sudan; ^3^ Faculty of Medicine Sudan University of Science and Technology Khartoum Sudan; ^4^ Al‐Neelain University Faculty of Medicine Khartoum Sudan; ^5^ Department of Medicine Fatima Memorial Hospital College of Medicine and Dentistry Lahore Pakistan; ^6^ Department of Medicine Nishtar Medical University Multan Pakistan; ^7^ Allama Iqbal Medical College Lahore Pakistan

## Abstract

**Background:**

Cerebrovascular disease (CVD) remains a leading cause of death, with obesity exacerbating stroke risk through multiple metabolic pathways. However, long‐term trends in CVD‐related mortality among obese adults in the United States remain inadequately defined.

**Methods:**

We analyzed national mortality data from 1999 to 2020 using the CDC WONDER database. Deaths were included if CVD (ICD‐10 I60–I69) was the underlying cause and obesity (E66) a contributing cause. Age‐adjusted mortality rates (AAMRs) were calculated, and temporal trends were evaluated using Joinpoint regression to estimate annual percent change (APC) and average annual percent change (AAPC).

**Results:**

From 1999 to 2020, 26,410 CVD‐related deaths occurred among obese adults. The overall AAMR was 0.53 per 100,000, with an AAPC of 4.59% (95% CI: 3.94 to 5.24). A statistically significant change in trend slope was observed after 2008, with accelerated mortality increases. Females had higher AAMRs (0.56) than males (0.49), though males experienced steeper increases (AAPC 5.98% vs. 3.64%). American Indian/Alaska Native and Black adults had the highest AAMRs (1.11 and 1.01, respectively). Mortality increased in all racial/ethnic groups, most rapidly among White individuals (AAPC 4.66%). Non‐metropolitan areas showed higher mortality than metropolitan areas (0.71 vs. 0.50), with a widening urban–rural gap. Regionally, the West and Midwest had the highest AAMRs (0.59 and 0.57, respectively). Mortality rose across all age groups, with the steepest increases in younger adults aged 25–54 years. Most deaths occurred in hospitals (56%), followed by home (22.8%) and nursing facilities (15.7%).

**Conclusions:**

CVD‐related mortality among obese adults has increased significantly since 1999, with substantial disparities across sex, race, geography, and age, highlighting the need for focused public health strategies.

## Graphical abstract

Using U.S. CDC WONDER mortality data from 1999–2020, cerebrovascular disease–related mortality among adults with obesity increased steadily, with acceleration after 2008. Pronounced disparities were observed by sex, race/ethnicity, geography, and age, with the fastest increases among younger adults.

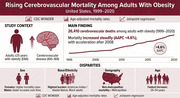

**Abbreviations**
AAPC: Average Annual Percent Change
AAMR: Age‐Adjusted Mortality Rate
AI/AN: American Indian or Alaska Native
APC: Annual Percent Change
BMI: Body Mass Index
CDC: Centers for Disease Control and Prevention
CI: Confidence Interval
CVD: Cerebrovascular Disease
CVA: Cerebrovascular Accident
ICD‐10: International Classification of Diseases, 10th Revision
IRB: Institutional Review Board
NHANES: National Health and Nutrition Examination Survey
NCHS: National Center for Health Statistics
REGARDS: Reasons for Geographic and Racial Differences in Stroke
STROBE: Strengthening the Reporting of Observational Studies in Epidemiology
U.S.: United States
WONDER: Wide‐ranging Online Data for Epidemiologic Research

## 1 Introduction

Cerebrovascular disease (CVD), including stroke, remains a major cause of morbidity and mortality in the United States and globally, particularly among individuals with modifiable cardiovascular risk factors such as obesity (Martin et al., 2024; Benjamin et al., 2019). Obesity has long been implicated in the development of vascular diseases through pathways involving chronic inflammation, atherosclerosis, and endothelial dysfunction (Lavie et al., 2015). Importantly, central adiposity—rather than general body mass—has been more strongly associated with ischemic stroke, reflecting the pathogenic role of visceral fat (Suk et al., 2003).

Despite improvements in stroke prevention and acute management, disparities in cerebrovascular mortality persist. In the United States, Black and American Indian or Alaska Native individuals experience disproportionately higher rates of stroke mortality, particularly when obesity is present (Carnethon et al., 2017). These disparities reflect not only biological interactions between race, adiposity, and vascular risk, but also broader structural factors, including access to care, socioeconomic status, and health literacy. Moreover, geographic variation further complicates the landscape, with higher stroke mortality rates consistently reported in the southeastern “Stroke Belt” (Howard et al., 2005).

Recent epidemiological studies indicate a troubling rise in obesity‐related cardiovascular deaths over the past two decades (Patel et al., 2015). While stroke mortality has declined in the general population due to improvements in prevention and acute management, rising obesity prevalence may attenuate population‐level gains by contributing to an increased burden of cerebrovascular risk factors, particularly among younger adults (Guo et al., 2016).

Despite these trends, limited research has focused specifically on long‐term national trends in CVD‐related mortality among obese adults, especially with stratification by demographic and geographic subgroups. Understanding these patterns is crucial for developing targeted, evidence‐based interventions to reduce disparities and prevent premature death.

## 2 Methods

### 2.1 Study Design

We conducted a cross‐sectional analysis of U.S. national mortality data using the Centers for Disease Control and Prevention (CDC) Wide‐ranging Online Data for Epidemiologic Research (WONDER) platform (Centers for Disease Control and Prevention (CDC) 2020). This publicly accessible database contains de‐identified death certificate records for all U.S. residents. Our study period spanned from 1999 through 2020 and focused on adults aged 25 years and older in whom CVD was listed as the underlying cause of death and obesity was documented as a contributing cause.

Causes of death were identified using the International Classification of Diseases, Tenth Revision (ICD‐10) (Free 2019 ICD‐10‐CM Codes 2019). CVD was defined using ICD‐10 codes I60–I69 and was required to be listed as the underlying cause of death. Obesity was defined by code E66 and included as a contributing cause. We included only deaths in which both conditions were jointly reported (I60–I69 as the underlying cause and E66 as a contributing cause) (Supplementary Table S1).

Because CDC WONDER data are publicly available and de‐identified, this study did not require Institutional Review Board (IRB) approval. The study was conducted and reported in accordance with the Strengthening the Reporting of Observational Studies in Epidemiology (STROBE) guidelines (von Elm et al., 2008) (Supplementary Table S2).

### 2.2 Data Extraction

We extracted national mortality data for adult decedents and conducted subgroup analyses stratified by sex, race/ethnicity, ten‐year age intervals (from 25–34 up to ≥85 years), urbanization level (metropolitan vs. non‐metropolitan), U.S. Census region (Northeast, Midwest, South, West), U.S. state of residence, and place of death.

Race and ethnicity classifications were based on information recorded on death certificates, typically reported by the funeral director based on next‐of‐kin input. Urbanization was categorized using the 2013 National Center for Health Statistics (NCHS) Urban–Rural Classification Scheme, defining metropolitan areas as those with ≥50,000 residents and non‐metropolitan areas as those with smaller populations (Ingram and Franco, 2014).

To protect confidentiality and ensure valid estimates, CDC WONDER suppresses data when mortality counts are fewer than 10. We limited analyses to strata and years where suppression did not occur, especially in smaller subpopulations such as racial/ethnic minorities and younger age groups.

### 2.3 Statistical Analysis

We retrieved annual mortality counts and corresponding population estimates from CDC WONDER. Age‐adjusted mortality rates (AAMRs) were computed using the direct method and standardized to the 2000 U.S. standard population. Rates were reported per 100,000 individuals with 95% confidence intervals (CIs) calculated assuming a Poisson distribution.

To evaluate temporal trends, we applied Joinpoint regression analysis. Log‐linear segmented regression models estimated the Annual Percent Change (APC) for each segment and the Average Annual Percent Change (AAPC) across the full study period. Joinpoints were identified using a Monte Carlo permutation method, with statistical significance defined as a two‐sided *P* < 0.05.

Trend analysis was conducted using the Joinpoint Regression Program (version 5.0.2) from the U.S. National Cancer Institute (National Cancer Institute 2023). Models started with 0 joinpoints and sequentially added joinpoints based on significance testing (Kim et al., 2000). Separate joinpoint models were generated for each subgroup, including sex, race/ethnicity, age group, region, and urbanization level. All APCs and corresponding 95% CIs were reported for each segment.

We performed between‐group comparisons to identify disparities in mortality burden and trend trajectories. Tests for parallelism assessed whether the shape and direction of trends differed significantly across strata. Supplementary analyses and data visualizations were performed using Python (version 3.10) with the statsmodels, matplotlib, and seaborn libraries.

All *p*‐values reported for annual percent changes (APCs) and average annual percent changes (AAPCs) were derived from Joinpoint regression analyses, while supplementary descriptive analyses and data visualizations were conducted using Python.

## 3 Results

### 3.1 Overall National Trends

Between 1999 and 2020, a total of 26,410 deaths were attributed to CVD among obese adults in the United States. The overall age‐adjusted mortality rate (AAMR) for this population was 0.53 per 100,000 (95% CI: 0.45 to 0.62) (Table 1).

**TABLE 1:** Demographic characteristics of decedents with cerebrovascular disease (CVD)–Related mortality among adults with obesity, United States, 1999–2020.

Characteristics	Deaths (%)	AAMR (95% CI) per 100 000
**Entire Cohort**	26,410 (100%)	0.53 (95% CI: 0.45 to 0.62)
**Gender**		
Female	15,033 (56.9%)	0.56 (95% CI: 0.49 to 0.64)
Male	11,377 (43.1%)	0.49 (95% CI: 0.40 to 0.58)
**Census Region**		
Northeast	3522 (13.3%)	5.58 (95% CI: 4.77 to 6.39)
Midwest	6216 (23.5%)	4.34 (95% CI: 3.64 to 5.04)
South	10,261 (38.9%)	4.40 (95% CI: 3.59 to 5.21)
West	6411 (24.3%)	4.04 (95% CI: 3.38 to 4.69)
**Race/Ethnicity**		
NH American Indian or Alaska Native	222 (0.8%)	1.11 (95% CI: 0.43 to 1.19)
NH Asian or Pacific Islander	376 (1.4%)	0.23 (95% CI: 0.18 to 0.28)
NH Black or African American	5,242 (19.8%)	1.01 (95% CI: 0.87 to 1.14)
NH White	20,570 (77.9%)	0.49 (95% CI: 0.42 to 0.57)
Hispanic or Latino	2009 (7.6%)	0.42 (95% CI: 0.35 to 0.50)
**Urbanization**		
Metropolitan (Urban)	20,502 (77.6%)	0.50 (95% CI: 0.42 to 0.58)
Non‐metropolitan (Rural)	5908 (22.4%)	0.71 (95% CI: 0.61 to 0.82
Ten‐Year Age Groups*		
25–34 years	472 (1.8%)	0.06 (95% CI: 0.05 to 0.07)
35–44 years	1608 (6.1%)	0.18 (95% CI: 0.15 to 0.21)
45–54 years	3704 (14.0%)	0.40 (95% CI: 0.34 to 0.46)
55–64 years	6229 (23.6%)	0.78 (95% CI: 0.67 to 0.89)
65–74 years	6886 (26.1%)	1.28 (95% CI: 1.10 to 1.46)
75–84 years	5423 (20.5%)	1.77 (95% CI: 1.49 to 2.05)
85+ years	2088 (7.9%)	1.69 (95% CI: 1.46 to 1.92)
**Place of Death†**		
Medical facility	14,789 (56.0%)	—
Decedent's home	6033 (22.8%)	—
Hospice facility	677 (2.6%)	—
Nursing home/Long‐term care facility	4140 (15.7%)	—

**Abbreviations**: AAMR = age adjusted mortality rate, NH = non‐Hispanic.

*Crude mortality rate (CR) is used for analysis instead of age adjusted mortality rates (AAMR) for Age groups.

†Age Adjusted Mortality Rates (AAMRs) is not applicable for Place of Death.

**FIGURE 1:**
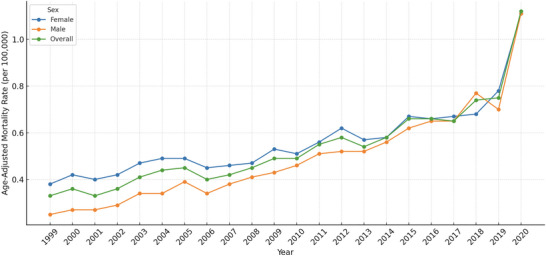
Trends in cerebrovascular disease (CVD)–related mortality among adults with obesity, stratified by sex and overall, United States, 1999–2020.

Joinpoint regression identified two significant trend segments. From 1999 to 2008, the AAMR increased at an average annual percentage change (APC) of 3.43% (95% CI: 1.90 to 4.99; *P* = 0.0022). This was followed by an accelerated increase from 2009 to 2020, with an APC of 5.87% (95% CI: 4.06 to 7.70; *P* = 0.0001). Over the full study period, the average annual percent change (AAPC) was 4.59% (95% CI: 3.94 to 5.24; *P* < 0.0001), confirming a statistically significant and persistent rise in CVD‐related mortality among obese adults (Table 2; Figure 1).

**TABLE 2:** Annual percentage changes (APCs) and average annual percentage changes (AAPCs) in cerebrovascular disease (CVD)–Related mortality among adults with obesity, United States, 1999–2020.

Characteristics	Trend segment	Year interval	APC (95% CI)	AAPC (95% CI)	*P*‐value
**Entire Cohort**				4.59 (3.94 to 5.24)	< 0.0001
	1	1999–2008	3.43 (1.90 to 4.99)		0.0022
	2	2009–2020	5.87 (4.06 to 7.70)		0.0001
**Gender**					
Female				3.64 (2.97 to 4.32)	< 0.0001
	1	1999–2008	2.23 (0.93 to 3.56)		0.0099
	2	2009–2020	5.12 (3.24 to 7.04)		0.0003
Male				5.98 (5.39 to 6.57)	< 0.0001
	1	1999–2008	5.53 (4.17 to 6.91)		< 0.0001
	2	2009–2020	6.86 (5.11 to 8.64)		< 0.0001
**Census Region**					
Northeast				5.58 (4.77 to 6.39)	< 0.0001
	1	1999–2008	4.18 (2.26 to 6.14)		0.0026
	2	2009–2020	5.97 (3.55 to 8.44)		0.0006
Midwest				4.34 (3.64 to 5.04)	< 0.0001
	1	1999–2008	3.13 (1.10 to 5.20)		0.0162
	2	2009–2020	6.20 (4.66 to 7.76)		< 0.0001
South				4.40 (3.59 to 5.21)	< 0.0001
	1	1999–2008	3.62 (2.40 to 4.86)		0.0002
	2	2009–2020	6.01 (4.85 to 7.19)		< 0.0001
West				4.04 (3.38 to 4.69)	< 0.0001
	1	1999–2008	4.39 (2.43 to 6.39)		0.0013
	2	2009–2020	5.78 (4.23 to 7.36)		<0.0001
**Race/Ethnicity**					
NH American Indian or Alaska Native				1.93 (−1.13 to 5.10)	0.1836
	1	1999–2008	1.65 (−4.41 to 8.14)		0.5453
	2	2009–2020	2.21 (−2.38 to 7.01)		0.3137
NH Asian or Pacific Islander				5.14 (−0.34 to 10.93)	0.1163
	1	1999–2020	5.14 (−0.34 to 10.93)		0.1163
NH Black or African American				3.50 (2.53 to 4.48)	< 0.0001
	1	1999–2008	3.91 (1.31 to 6.59)		0.0180
	2	2009–2020	5.17 (2.49 to 7.92)		0.0033
NH White				4.66 (4.03 to 5.28)	< 0.0001
	1	1999–2008	2.71 (1.86 to 3.57)		0.0002
	2	2009–2020	6.39 (4.80 to 7.99)		< 0.0001
Hispanic or Latino				3.32 (0.94 to 5.76)	< 0.0001
	1	1999–2008	2.42 (−1.56 to 6.56)		0.2706
	2	2009–2020	4.28 (0.72 to 7.96)		0.0394
**Urbanization**					
Metropolitan (Urban)				4.91 (4.27 to 5.56)	< 0.0001
	1	1999–2008	3.76 (2.39 to 5.14)		0.0006
	2	2009–2020	6.11 (4.27 to 7.99)		0.0001
Non‐metropolitan (Rural)				4.06 (3.07 to 5.06)	< 0.0001
	1	1999–2008	2.52 (−0.46 to 5.58)		0.137
	2	2009–2020	6.15 (3.76 to 8.60)		0.0004
Ten‐Year Age Groups*^§^					
25–34 years				6.07 (3.82 to 8.36)	< 0.0001
	1	1999–2008	4.71 (1.93 to 7.55)		0.0121
	2	2009–2020	17.26 (−15.81 to 63.32)		0.519
35–44 years				6.15 (5.45 to 6.85)	< 0.0001
	1	1999–2008	5.25 (3.34 to 7.2)		0.0006
	2	2009–2020	6.06 (4.04 to 8.12)		0.0001
45–54 years				4.95 (4.28 to 5.63)	< 0.0001
	1	1999–2008	5.31 (3.58 to 7.05)		0.0003
	2	2009–2020	6.22 (4.36 to 8.13)		0.0001
55–64 years				4.02 (3.31 to 4.74)	< 0.0001
	1	1999–2008	2.70 (1.15 to 4.27)		0.0089
	2	2009–2020	5.46 (3.44 to 7.51)		0.0003
65–74 years				4.09 (3.42 to 4.77)	< 0.0001
	1	1999–2008	3.29 (1.67 to 4.94)		0.0039
	2	2009–2020	5.40 (3.52 to 7.32)		0.0002
75–84 years				4.54 (3.67 to 5.42)	< 0.0001
	1	1999–2008	3.15 (1.07 to 5.28)		0.0174
	2	2009–2020	7.19 (5.14 to 9.28)		< 0.0001
85+ years				3.02 (1.73 to 4.32)	< 0.0001
	1	1999–2008	−2.54 (−5.71 to 0.72)		0.1642
	2	2009–2020	5.26 (2.74 to 7.84)		0.002

**Abbreviations**: AAMR = age adjusted mortality rate, AAPC = average annual percentage change, APC = annual percentage change, NH = non‐Hispanic.

*Crude rate is used for all age dependent analysis.

### 3.2 Trends by Sex

Between 1999 and 2020, a total of 26,410 CVD‐related deaths occurred among obese adults in the United States, including 15,033 (56.9%) among females and 11,377 (43.1%) among males. The average age‐adjusted mortality rate (AAMR) was higher among females, at 0.56 per 100,000 (95% CI: 0.49 to 0.64), compared to 0.49 per 100,000 (95% CI: 0.40 to 0.58) among males (Table 1).

Joinpoint regression identified two distinct temporal segments for each sex. Among females, the AAMR increased with an annual percent change (APC) of 2.23% (95% CI: 0.93 to 3.56, *p* = 0.0099) from 1999 to 2008, followed by a steeper rise of 5.12% (95% CI: 3.24 to 7.04, *p* = 0.0003) from 2009 to 2020. Among males, the corresponding APCs were 5.53% (95% CI: 4.17 to 6.91, *p* <0.0001) from 1999 to 2008 and 6.86% (95% CI: 5.11 to 8.64, *p* < 0.0001) from 2009 to 2020. Overall, the average annual percent change (AAPC) was 3.64% (95% CI: 2.97 to 4.32) in females and 5.98% (95% CI: 5.39 to 6.57) in males, reflecting a steeper long‐term increase in CVD mortality among obese males despite consistently higher rates in females (Table 2; Figure 1).

### 3.3 Trends by Race/Ethnicity

Between 1999 and 2020, pronounced racial and ethnic disparities were observed in CVD‐related mortality among obese adults in the United States. The highest age‐adjusted mortality rate (AAMR) was recorded among American Indian or Alaska Native individuals (1.11; 95% CI: 0.43 to 1.19), followed by Black adults (1.01; 95% CI: 0.87 to 1.14), White adults (0.49; 95% CI: 0.42 to 0.57), and Asian or Pacific Islanders (0.23; 95% CI: 0.18 to 0.28). The AAMR for Hispanic individuals was 0.42 (95% CI: 0.35 to 0.50) (Table 1).

**FIGURE 2:**
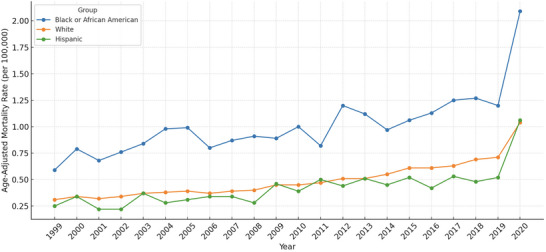
Trends in cerebrovascular disease (CVD)–related mortality among adults with obesity, stratified by race and ethnicity, United States, 1999–2020.

Joinpoint regression revealed varying temporal trends. Among White adults, mortality rates increased modestly from 1999–2008 (APC: 2.71%; 95% CI: 1.86 to 3.57) and accelerated thereafter from 2009 to 2020 (APC: 6.39%; 95% CI: 4.80 to 7.99), with an overall AAPC of 4.66% (95% CI: 4.03 to 5.28; *P* < 0.0001). Black adults experienced steady increases in both time segments (1999–2008 APC: 3.91%; 2009–2020 APC: 5.17%), with a significant AAPC of 3.50% (95% CI: 2.53 to 4.48). For Hispanic individuals, a moderate rise was noted from 1999 to 2008 (APC: 2.42%), followed by a sharper increase from 2009 to 2020 (APC: 4.28%; 95% CI: 0.72 to 7.96), resulting in an AAPC of 3.32% (95% CI: 0.94 to 5.76). Trends among Asian or Pacific Islanders were limited by smaller case numbers but still showed an upward trajectory (AAPC: 5.14%; 95% CI: −0.34 to 10.93). In contrast, American Indian or Alaska Native adults had less consistent patterns, with non‐significant increases in both intervals and a non‐significant AAPC of 1.93% (95% CI: −1.13 to 5.10) (Table 2; Figure 2).

### 3.4 Trends by Urbanization Status

Age‐adjusted mortality from CVD among obese adults was consistently higher in non‐metropolitan areas than in metropolitan ones. Between 1999 and 2020, the AAMR in non‐metropolitan areas was 0.71 per 100,000 (95% CI: 0.61 to 0.82), compared to 0.50 per 100,000 (95% CI: 0.42 to 0.58) in metropolitan areas (Table 1).

**FIGURE 3:**
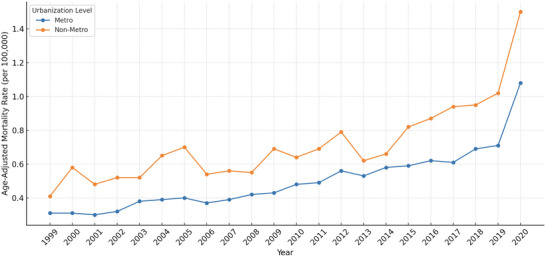
Trends in cerebrovascular disease (CVD)–related mortality among adults with obesity, stratified by urbanization level, United States, 1999–2020.

Joinpoint regression identified two distinct trend segments in both groups. In metropolitan areas, the annual percent change (APC) increased from 3.76% (95% CI: 2.39 to 5.14, *p* = 0.0006) during 1999–2008 to 6.11% (95% CI: 4.27 to 7.99, *p* = 0.0001) during 2009–2020. In non‐metropolitan areas, the APC rose from 2.52% (95% CI: −0.46 to 5.58, *p* = 0.137) to 6.15% (95% CI: 3.76 to 8.60, *p* = 0.0004) over the same periods. Overall, the average annual percent change (AAPC) was 4.91% (95% CI: 4.27 to 5.56) in metropolitan areas and 4.06% (95% CI: 3.07 to 5.06) in non‐metropolitan areas. (Table 2; Figure 3).

### 3.5 Trends by Census Region

Between 1999 and 2020, regional variation was observed in CVD‐related mortality among obese adults in the United States. The highest average age‐adjusted mortality rate (AAMR) was recorded in the West at 0.59 per 100,000 (95% CI: 0.51 to 0.66), followed by the Midwest at 0.57 (95% CI: 0.49 to 0.65), the South at 0.56 (95% CI: 0.48 to 0.65), and the Northeast, which had the lowest rate at 0.38 (95% CI: 0.31 to 0.44) (Table 1).

**FIGURE 4:**
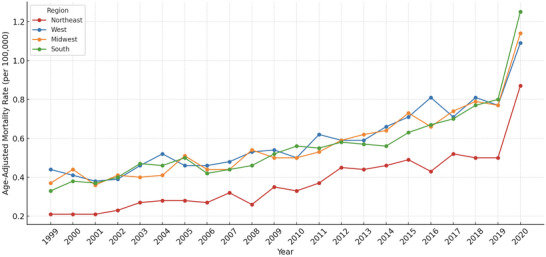
Trends in cerebrovascular disease (CVD)–related mortality among adults with obesity, stratified by U.S. Census region, United States, 1999–2020.

Joinpoint regression revealed a consistent two‐segment trend across all regions. In the Northeast, the annual percent change (APC) increased from 4.18% (95% CI: 2.26 to 6.14, *p* = 0.0026) during 1999–2008 to 5.97% (95% CI: 3.55 to 8.44, *p* = 0.0006) during 2009–2020. Similar upward shifts were observed in the Midwest, rising from 3.13% (95% CI: 1.10 to 5.20, *p* = 0.0162) to 6.20% (95% CI: 4.66 to 7.76, *p* < 0.0001); in the South, from 3.62% (95% CI: 2.40 to 4.86, p = 0.0002) to 6.01% (95% CI: 4.85 to 7.19, *p* < 0.0001); and in the West, from 4.39% (95% CI: 2.43 to 6.39, *p* = 0.0013) to 5.78% (95% CI: 4.23 to 7.36, *p* < 0.0001). The overall average annual percent change (AAPC) was highest in the Northeast at 5.58% (95% CI: 4.77 to 6.39), followed by the Midwest at 4.34% (95% CI: 3.64 to 5.04), the South at 4.40% (95% CI: 3.59 to 5.21), and the West at 4.04% (95% CI: 3.38 to 4.69). These results reflect a steady rise in mortality across all regions, with notable geographic disparities (Table 2; Figure 4).

### 3.6 Trends by State

Pronounced variation in CVD‐related mortality among obese adults was observed across U.S. states. The highest age‐adjusted mortality rates (AAMRs) were recorded in Vermont (1.21 per 100,000; 95% CI: 1.00 to 1.42), Oklahoma (1.10; 95% CI: 1.01 to 1.19), Minnesota (0.97; 95% CI: 0.90 to 1.03), Mississippi (0.92; 95% CI: 0.83 to 1.01), and Oregon (0.90; 95% CI: 0.82 to 0.97).

**FIGURE 5:**
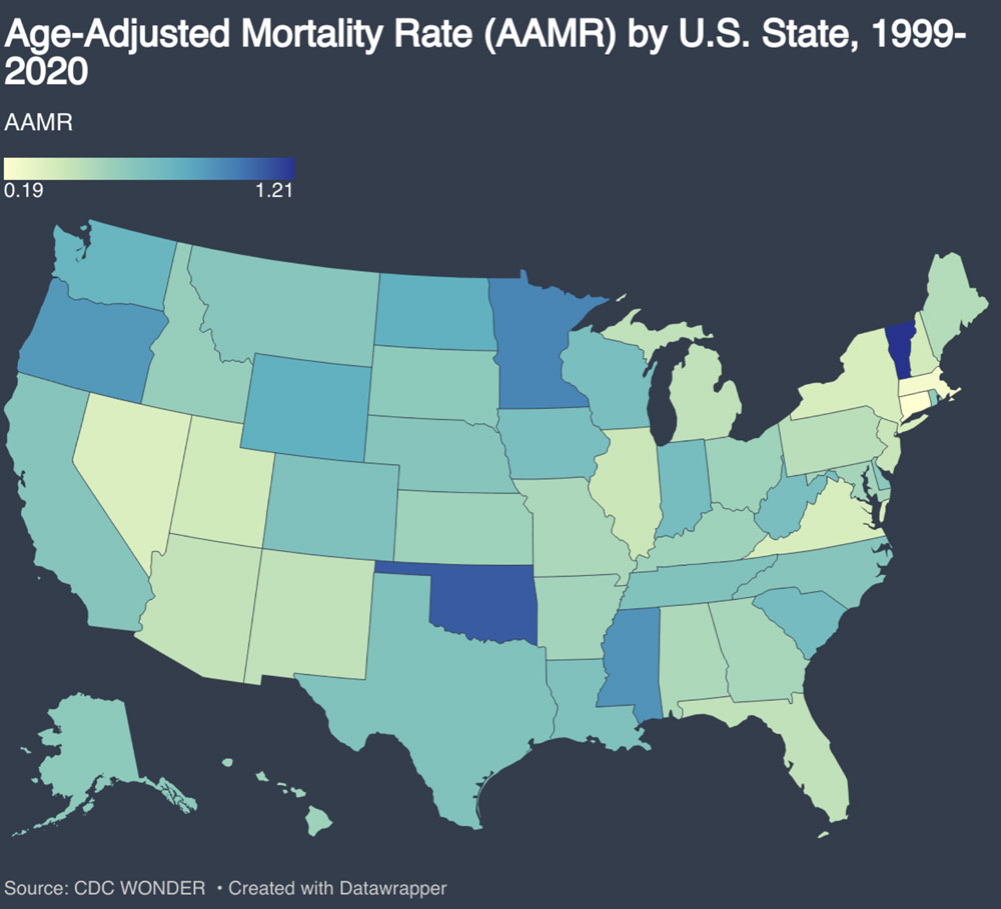
Geographic distribution of cerebrovascular disease (CVD)–related mortality among adults with obesity by state, United States, 1999–2020.

In contrast, the lowest AAMRs were seen in New York (0.35; 95% CI: 0.33 to 0.37), Virginia (0.35; 95% CI: 0.32 to 0.39), Nevada (0.34; 95% CI: 0.28 to 0.40), Massachusetts (0.23; 95% CI: 0.20 to 0.26), and Connecticut (0.19; 95% CI: 0.16 to 0.23). There was a nearly sixfold difference in mortality between the highest and lowest burden states (Figure 5).

### 3.7 Trends by Ten‐Year Age Group

Between 1999 and 2020, CVD‐related mortality among obese adults increased across all age groups, with varying magnitudes of change. Age‐adjusted mortality rates (AAMRs) rose progressively with age, peaking in the 75–84 years group before slightly declining in the 85+ cohort. The AAMRs were lowest in young adults aged 25–34 years (0.06 per 100,000; 95% CI: 0.05 to 0.07) and increased steadily across older groups: 0.18 (95% CI: 0.15 to 0.21) in 35–44 years, 0.40 (95% CI: 0.34 to 0.46) in 45–54 years, 0.78 (95% CI: 0.67 to 0.89) in 55–64 years, 1.28 (95% CI: 1.10 to 1.46) in 65–74 years, and 1.77 (95% CI: 1.49 to 2.05) in 75–84 years, before slightly declining to 1.69 (95% CI: 1.46 to 1.92) in the 85+ group (Table 1).

**FIGURE 6:**
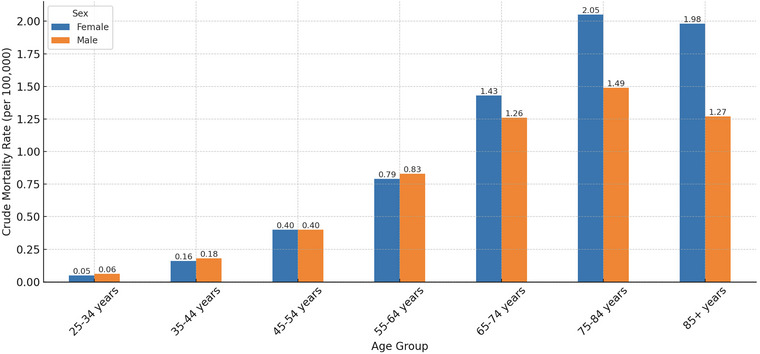
Crude mortality rates for cerebrovascular disease (CVD)–related deaths among adults with obesity, stratified by ten‐year age groups and sex, United States, 1999–2020.

Joinpoint regression identified two distinct temporal segments across most age groups: 1999–2008 and 2009–2020. Among adults aged 25–34 years, a modest increase from 1999 to 2008 (APC: 4.71%; 95% CI: 1.93 to 7.55; *P* = 0.0121) was followed by a steeper but non‐significant rise from 2009 to 2020 (APC: 17.26%; 95% CI: –15.81 to 63.32; *P* = 0.519), yielding an overall AAPC of 6.07% (95% CI: 3.82 to 8.36; *P* < 0.0001). Similarly, the 35–44 and 45–54 age groups experienced steady increases over both intervals, with AAPCs of 6.15% (95% CI: 5.45 to 6.85) and 4.95% (95% CI: 4.28 to 5.63), respectively. In middle‐aged adults, the AAPCs were slightly lower but remained statistically significant: 4.02% (95% CI: 3.31 to 4.74) in 55–64 years and 4.09% (95% CI: 3.42 to 4.77) in 65–74 years. The 75–84 year group showed a similar trend (AAPC: 4.54%; 95% CI: 3.67 to 5.42). Notably, the 85+ group was the only cohort to show a potential inflection in trend: a non‐significant decline from 1999 to 2008 (APC: –2.54%; 95% CI: –5.71 to 0.72; *P* = 0.1642) followed by a significant increase from 2009 to 2020 (APC: 5.26%; 95% CI: 2.74 to 7.84; *P* = 0.002), resulting in an overall AAPC of 3.02% (95% CI: 1.73 to 4.32). These results highlight a concerning increase in CVD‐related mortality across all age groups among obese adults, with the steepest relative rises observed in younger populations and sustained burdens among older adults. The age‐specific patterns suggest a dual challenge: emerging risk in early adulthood and persistent vulnerability in the elderly (Table 2; Figure 6).

### 3.8 Place of Death

Among the 26,410 recorded deaths, the majority occurred in medical facilities (inpatient settings), accounting for 56.0% of all deaths. A significant proportion also died at home (22.8%), followed by those in nursing homes or long‐term care facilities (15.7%). Additional deaths occurred in emergency departments or outpatient medical settings (9.7%), and a smaller fraction (2.6%) occurred in other or unspecified locations (Table 1).

## 4 Discussion

This national analysis reveals a significant and sustained rise in CVD‐related mortality among obese adults in the United States from 1999 to 2020. Over the 22‐year period, age‐adjusted mortality rates nearly doubled, with a statistically significant change in trend slope after 2008 indicating accelerated growth. The overall average annual percent change (AAPC) of 4.59% underscores a concerning upward trend in stroke‐related deaths in this vulnerable population. The acceleration in cerebrovascular mortality trends observed after 2008 likely reflects a convergence of epidemiologic and documentation‐related factors. These include rising prevalence of obesity and severe obesity, earlier accumulation of cardiometabolic risk factors, and improved clinical recognition of obesity as a contributor to adverse health outcomes. In addition, during the COVID‐19 pandemic, obesity was increasingly recognized as a major mortality risk factor, which may have led to more consistent documentation on death certificates and contributed to the sharper increases observed in the later years of the study period. (Virani et al., 2021; Ward et al., 2019).

Males experienced steeper long‐term increases despite females exhibiting higher absolute AAMRs. Racial and ethnic disparities were pronounced, with American Indian/Alaska Native (AI/AN) and Black adults bearing the highest stroke‐related mortality. Geographic variation was substantial, with elevated mortality in the Midwest, West, and non‐metropolitan areas. Notably, the most rapid relative increases occurred in younger adults (25–54 years), signaling a shift in stroke burden toward earlier life stages—an alarming trend for population health and workforce productivity (Yoon et al., 2015).

Although females exhibited higher overall AAMRs, the greater AAPC in males (5.98% vs. 3.64%) suggests a shifting sex‐based trend in CVD mortality among obese adults. This aligns with research indicating men may experience accelerated vascular aging due to increased visceral fat and androgen‐driven metabolic effects (Wajchenberg, 2000). Additionally, lower healthcare engagement and delayed stroke recognition among men may contribute to worse outcomes (Berglund et al., 2017). Conversely, women may face higher baseline stroke risk due to hormonal modulation, pregnancy‐related exposures, and comorbidities such as migraine and autoimmune disease (Rexrode et al., 2022; Bushnell et al., 2014). Despite this, they remain underrepresented in prevention trials and are less likely to receive acute interventions like thrombolysis or thrombectomy, exacerbating disparities (Labiche et al., 2002).

AI/AN and Black adults experienced the highest CVD‐related mortality, consistent with longstanding health inequities. AI/AN populations bear disproportionate burdens of obesity, hypertension, and diabetes, compounded by limited specialist access and underfunded tribal health systems (Espey et al., 2014; Warne and Lajimodiere, 2015). Black adults are affected by early‐onset hypertension, greater left ventricular hypertrophy, and reduced access to preventive stroke therapies (Benjamin et al., 2018). Although White adults had lower absolute mortality, their steep post‐2008 APC suggests increasing vulnerability, likely tied to rising severe obesity and rurality. Hispanic and Asian/Pacific Islander adults had lower baseline rates, potentially due to protective behaviors or genetics, though underrepresentation in stroke surveillance limits inference (Rodriguez et al., 2014).

CVD‐related AAMRs were persistently higher in non‐metropolitan areas, with a widening gap over time. These disparities likely stem from limited healthcare infrastructure, transportation barriers, workforce shortages, and delayed emergency response in rural settings (Moy et al., 2017). Residents of these areas are less likely to receive primary prevention or timely acute care, such as thrombolysis (Tong et al., 2021). Rural regions, particularly in the South and Appalachia, also show higher obesity prevalence. Environmental risk factors, including lack of recreational spaces, food deserts, and greater pollutant exposure, further contribute to elevated stroke risk (Befort et al., 2012).

Marked geographic disparities emerged, with the highest mortality rates in the states of West and Midwest, especially in Oklahoma and Mississippi, with high obesity prevalence and limited public health investment (Trust for America's Health 2020; Levi et al., 2014). Although the Western United States has historically demonstrated lower overall obesity prevalence in population‐level analyses, this region is characterized by substantial heterogeneity. Several Western states include large rural or frontier populations with limited access to preventive healthcare and acute stroke services, as well as rising rates of severe obesity in specific subgroups. These factors may offset regional advantages observed in aggregate obesity metrics and contribute to higher obesity‐associated cerebrovascular mortality in this analysis. The well‐known “stroke belt” in the Southeastern U.S. remains a high‐risk zone due to lifestyle factors, socioeconomic deprivation, and poor access to specialized care (Howard et al., 2007). Conversely, the Northeast had the lowest AAMRs but the highest AAPC, indicating rising risk from a low baseline. States like Massachusetts, New York, and Connecticut may benefit from stronger stroke systems, robust preventive infrastructure, and Medicaid expansion (Ikeme et al., 2022).

The rising cerebrovascular mortality observed in obese adults aligns with extensive literature identifying obesity as a potent risk factor for stroke. Excess body mass index (BMI) is consistently linked to increased risk of both ischemic and hemorrhagic stroke via mechanisms such as hypertension, diabetes, dyslipidemia, and systemic inflammation (Strazzullo et al., 2010; Wang et al., 2005; Jin et al., 2023). A meta‐analysis of over 2 million individuals from Asia and Europe confirmed that obesity significantly elevates stroke mortality risk, especially among younger populations (Song et al., 2004). Our results also echo reports of stagnation or even reversal in U.S. stroke mortality trends since the early 2010s. Martin et al. attributed this pattern to worsening obesity, healthcare disparities, and inadequate control of modifiable risk factors (Martin et al., 2025). NHANES data similarly highlight poor blood pressure control and underuse of statins and antihypertensives among obese individuals (Mozaffarian et al., 2016). The sharp post‐2008 increase in mortality may reflect the rise of severe obesity (BMI ≥ 40 kg/m^2^), projected to affect nearly 25% of U.S. adults by 2030 (Ward et al., 2017). Severe obesity is linked to prothrombotic states, left atrial enlargement, and carotid intima‐media thickening, all contributors to cerebrovascular risk (Quiñones‐Ossa et al., 2021; Kotsis et al., 2010).

Sex‐based differences in our findings partly align with existing studies. While men typically have higher stroke incidence and fatality, research suggests obese women may face heightened risk due to hormonal factors, fat distribution, and reduced physical activity (Rexrode et al., 1997; Skolarus et al., 2014). The INTERSTROKE study, for example, found central obesity more strongly associated with stroke in women even after adjusting for traditional risk factors (O'Donnell et al., 2010). Racial disparities, particularly among AI/AN and Black adults, are supported by prior evidence. Black individuals often experience earlier hypertension, more severe disease progression, and less access to evidence‐based treatment (Aradine et al., 2022; Cruz‐Flores et al., 2011). AI/AN populations face similar structural barriers, including food insecurity and limited healthcare access (Breathett et al., 2020). Notably, previous studies have rarely stratified stroke mortality by obesity status, making this analysis a unique and valuable contribution. Our geographic findings are also consistent with the REGARDS (Reasons for Geographic and Racial Differences in Stroke) study, which identified the Southeastern “stroke belt” as a high‐mortality region, particularly in rural and low‐income communities (Howard et al., 2011). Elevated mortality in Mississippi and Oklahoma aligns with known gaps in access to neurologists and stroke centers (Hale et al., 2010). These patterns reflect complex interactions among environment, health policy, social determinants, and healthcare infrastructure.

### 4.1 Strengths and Limitations

This study offers several important strengths. It is the first to systematically examine national trends in CVD‐related mortality specifically among obese adults in the United States using a 22‐year longitudinal dataset. The use of CDC WONDER, a validated and comprehensive vital statistics registry, enhances the generalizability of findings across diverse demographic and geographic strata. Furthermore, stratified Joinpoint regression analysis allowed for detailed assessment of time‐segmented trends across sex, race/ethnicity, urbanization, age, and region, revealing nuanced epidemiologic patterns often obscured in aggregate data.

The relatively low absolute age‐adjusted mortality rates observed in this study should be interpreted in the context of well‐documented underreporting of obesity on death certificates. Obesity is inconsistently recorded as a contributing cause of death, particularly when it is not perceived as directly related to the terminal event. Consequently, our findings likely represent a conservative subset of cerebrovascular deaths in which obesity was formally documented and may underestimate the true cerebrovascular mortality burden attributable to obesity at the population level.

However, several limitations should be noted. First, the reliance on death certificate data introduces potential misclassification bias, particularly in determining both CVD and obesity as contributing causes of death. Obesity is often underreported or inconsistently coded on death certificates, possibly leading to underestimation of the true burden. Second, the study cannot differentiate between stroke subtypes (e.g., ischemic vs. hemorrhagic) due to the use of grouped ICD‐10 codes, which may mask differences in pathophysiology and clinical trajectories. Third, residual confounding by unmeasured variables such as socioeconomic status, access to care, medication adherence, and lifestyle behaviors is possible, particularly when examining disparities across regions and subgroups. Last, given the ecological design, the study cannot establish causality, nor can it track individuallevel risk factor trajectories over time.

This analysis was designed to evaluate temporal trends in cerebrovascular mortality when obesity was documented as a contributing cause of death, rather than to assess the presence of obesity across all rankable causes of mortality, which represents a distinct research question beyond the scope of the current study.

## 5 Conclusion

The sustained rise in CVD mortality among obese adults in the United States represents a critical and underrecognized shift in the epidemiology of stroke. This study reveals not only a growing national burden but also sharp disparities by sex, race, geography, and age, suggesting that stroke prevention efforts have failed to adequately reach populations most affected by obesity. The accelerated mortality increases observed after 2008, particularly among younger adults and non‐metropolitan communities, highlight a troubling reversal of prior gains in stroke control.

Moving forward, obesity must be treated not only as a cardiovascular risk factor but also as a central determinant in cerebrovascular health policy and clinical guidelines. Public health frameworks should target high‐risk subgroups with culturally and geographically tailored interventions, while healthcare systems must address structural barriers to early detection, risk modification, and post‐stroke care in obese populations. Without urgent and focused efforts, the dual epidemic of obesity and CVD is poised to deepen existing health inequities and strain already vulnerable care systems.

These findings provide actionable insight for policymakers and healthcare providers by identifying demographic and geographic groups at disproportionate risk. Targeted prevention strategies focusing on obesity management, blood pressure control, and early cerebrovascular risk reduction, particularly among Black and American Indian or Alaska Native populations and residents of non‐metropolitan regions, may help mitigate widening disparities in cerebrovascular mortality.

## Author Contributions

**Ibrahim Nagmeldin Hassan**: conceptualization, methodology, project administration, visualization, writing – original draft, and writing – review and editing. **Siddig Yaqub**: project administration, validation, writing – original draft, writing – review and editing. **Muhsin Ibrahim**: writing – original draft, writing – review and editing. **Nagmeldin Abuassa**: writing – original draft, writing – review and editing. **Mohamed Ibrahim**: conceptualization, formal analysis, visualization, writing – original draft, writing – review and editing. **Shahzaib Ahmed**: project administration, validation, writing – review and editing. **Allahdad Khan**: writing – original draft, writing – review and editing. **Hamza Ashraf**: formal analysis, visualization, writing – original draft, writing – review and editing. All authors read and approved the final manuscript.

## Funding

The authors have nothing to report.

## Conflicts of Interest

The authors declare no conflicts of interest.

## Supporting information




**Supplementary TABLE S1** ICD‐10 codes used to define cerebrovascular disease (CVD)–related mortality among adults with obesity, with inclusion and exclusion criteria.
**Supplementary TABLE S2** STROBE checklist for reporting observational studies using routinely collected mortality data.

## Data Availability

The data that support the findings of this study are openly available in CDC WONDER at https://wonder.cdc.gov/, reference number N/A.
