# Supplementary material for: National Trends in Cerebrovascular Disease–Related Mortality among Adults With Obesity in the United States, 1999–2020
Source: Brain Behav. 2026 Feb 28;16(3):e71276. doi: 10.1002/brb3.71276 (PMC12949721; doi:10.1002/brb3.71276)
Supplement: Supplementary file 1 — Supplementary TABLE S1 ICD‐10 codes used to define cerebrovascular disease (CVD)–related mortality among adults with obesity, with inclusion and exclusion criteria. Supplementary TABLE S2 STROBE checklist for reporting observational studies using routinely collected mortality data. [file BRB3-16-e71276-s001.docx]

Supplementary Materials

**Supplementary Table S1. ICD-10 codes used to define cerebrovascular disease (CVA)–related mortality among adults with obesity, with inclusion and exclusion criteria.**

| **Characteristics** | **ICD-10 Code(s)** | **Description** | **Use in Study** | **Justification** |
| --- | --- | --- | --- | --- |
| **Cerebrovascular Disease (CVA)** |  |  |  |  |
|  | I60–I69 | Cerebrovascular diseases | Included as underlying cause of death | Encompasses ischemic stroke, hemorrhagic stroke, and sequelae of cerebrovascular disease |
| **Obesity** |  |  |  |  |
|  | E66 | Obesity | Included as contributing cause of death | Reflects obesity as a comorbid condition influencing CVA mortality |
| **Excluded Codes** |  |  |  |  |
|  | R41, G45, G46 | Cognitive symptoms, transient ischemic attacks | Excluded | Not specific to fatal CVA events or not coded as causes of death |

**ICD-10 = International Classification of Diseases, 10th Revision; CVA = cerebrovascular accident. Only ICD-10 codes listed as the underlying cause of death (for CVA) or as a contributing cause of death (for obesity) were included in the study dataset.**

**Table S2. Strengthening the Reporting of Observational Studies in Epidemiology (STROBE) Checklist**

|  | **Item No** | **Recommendation** | **Section or**  **page number** |
| --- | --- | --- | --- |
| **Title and abstract** | 1 | (*a*) Indicate the study’s design with a commonly used term in  the title or the abstract | 1 |
|  |  | (*b*) Provide in the abstract an informative and balanced | 2 |
|  |  | summary of what was done and what was found |  |
| **Introduction** |  |  |  |
| Background/rationale | 2 | Explain the scientific background and rationale for the | 3 |
|  |  | investigation being reported |  |
| Objectives | 3 | State specific objectives, including any prespecified | 3 |
|  |  | hypotheses |  |
| **Methods** |  |  |  |
| Study design | 4 | Present key elements of study design early in the paper | 4 |
| Setting | 5 | Describe the setting, locations, and relevant dates, including | 4 |
|  |  | periods of recruitment, exposure, follow-up, and data |  |
|  |  | collection |  |
| Participants | 6 | (*a*) Give the eligibility criteria, and the sources and methods | 4 |
|  |  | of selection of participants |  |
| Variables | 7 | Clearly define all outcomes, exposures, predictors, potential | 4 |
|  |  | confounders, and effect modifiers. Give diagnostic criteria, if |  |
|  |  | applicable |  |
| Data sources/ | 8* | For each variable of interest, give sources of data and details | 4 |
| measurement |  | of methods of assessment (measurement). Describe |  |
|  |  | comparability of assessment methods if there is more than |  |
|  |  | one group |  |
| Bias | 9 | Describe any efforts to address potential sources of bias | 4 |
| Study size | 10 | Explain how the study size was arrived at | 4 |
| Quantitative variables | 11 | Explain how quantitative variables were handled in the | 4 |
|  |  | analyses. If applicable, describe which groupings were |  |
|  |  | chosen and why |  |
| Statistical methods | 12 | (*a*) Describe all statistical methods, including those used to | 5 |
|  |  | control for confounding |  |
|  |  | (*b*) Describe any methods used to examine subgroups and | 5 |
|  |  | interactions |  |
|  |  | (c) Explain how missing data were addressed | 5 |
|  |  | (*d*) If applicable, describe analytical methods taking account | Not applicable |
|  |  | of sampling strategy |  |
|  |  | (*e*) Describe any sensitivity analyses | Not applicable |
| **Results** |  |  |  |
| Participants | 13* | (a) Report numbers of individuals at each stage of study—eg | 5 |
|  |  | numbers potentially eligible, examined for eligibility, |  |
|  |  | confirmed eligible, included in the study, completing follow- |  |
|  |  | up, and analysed |  |
|  |  | (b) Give reasons for non-participation at each stage | 5 |
|  |  | (c) Consider use of a flow diagram | - |
| Descriptive data | 14* | (a) Give characteristics of study participants (eg | 5-8 |
|  |  | demographic, clinical, social) and information on exposures |  |
|  |  | and potential confounders |  |

|  |  | (b) Indicate number of participants with missing data for  each variable of interest | 5-8 |
| --- | --- | --- | --- |
| Outcome data | 15* | Report numbers of outcome events or summary measures | 5-8 |
| Main results | 16 | (*a*) Give unadjusted estimates and, if applicable, confounder- adjusted estimates and their precision (eg, 95% confidence  interval). Make clear which confounders were adjusted for and why they were included | 5-8 |
|  |  | (*b*) Report category boundaries when continuous variables were categorized | Not applicable |
|  |  | © If relevant, consider translating estimates of relative risk  into absolute risk for a meaningful time period | Not applicable |
| Other analyses | 17 | Report other analyses done—eg analyses of subgroups and  interactions, and sensitivity analyses | Not applicable |
| **Discussion** |  |  |  |
| Key results | 18 | Summarise key results with reference to study objectives | 9 |
| Limitations | 19 | Discuss limitations of the study, taking into account sources of potential bias or imprecision. Discuss both direction and  magnitude of any potential bias | 10-11 |
| Interpretation | 20 | Give a cautious overall interpretation of results considering objectives, limitations, multiplicity of analyses, results from  similar studies, and other relevant evidence | 10-11 |
| Generalisability | 21 | Discuss the generalisability (external validity) of the study  results | 10-11 |
| **Other information** |  |  |  |
| Funding | 22 | Give the source of funding and the role of the funders for the present study and, if applicable, for the original study on  which the present article is based | 12 |
